# Human Rabies — Utah, 2018

**DOI:** 10.15585/mmwr.mm6905a1

**Published:** 2020-02-07

**Authors:** Dallin Peterson, Bree Barbeau, Keegan McCaffrey, Randon Gruninger, Jeffrey Eason, Cindy Burnett, Angela Dunn, Melissa Dimond, Jesse Harbour, Alessandro Rossi, Bert Lopansri, Kristin Dascomb, Tara Scribellito, TaLeah Moosman, Louise Saw, Curtis Jones, Michael Belenky, Lily Marsden, Michael Niezgoda, Crystal M. Gigante, Rene Edgar Condori, James A. Ellison, Lillian A. Orciari, Pamela Yager, Jesse Bonwitt, Erin R. Whitehouse, Ryan M Wallace

**Affiliations:** ^1^Utah Department of Health, Salt Lake City; ^2^Intermountain Healthcare, Salt Lake City, Utah; ^3^Salt Lake County Health Department, Salt Lake City, Utah; ^4^Central Utah Public Health Department, Nephi; ^5^Utah County Health Department, Provo; ^6^Division of High-Consequence Pathogens and Pathology, National Center for Emerging and Zoonotic Infectious Diseases, CDC; ^7^Oak Ridge Institute for Science and Education, Oak Ridge, Tennessee; ^8^Epidemic Intelligence Service, CDC.

On November 3, 2018, the Utah Department of Health (UDOH) was notified of a suspected human rabies case in a man aged 55 years. The patient’s symptoms had begun 18 days earlier, and he was hospitalized for 15 days before rabies was suspected. As his symptoms worsened, he received supportive care, but he died on November 4. On November 7, a diagnosis of rabies was confirmed by CDC. This was the first documented rabies death in a Utah resident since 1944. This report summarizes the patient’s clinical course and the subsequent public health investigation, which determined that the patient had handled several bats in the weeks preceding symptom onset. Public health agencies, in partnership with affected health care facilities, identified and assessed the risk to potentially exposed persons, facilitated receipt of postexposure prophylaxis (PEP), and provided education to health care providers and the community about the risk for rabies associated with bats. Human rabies is rare and almost always fatal. The findings from this investigation highlight the importance of early recognition of rabies, improved public awareness of rabies in bats, and the use of innovative tools after mass rabies exposure events to ensure rapid and recommended risk assessment and provision of PEP.

## Case Report

On October 17 and 18, 2018, a man aged 55 years who lived in Utah sought chiropractic treatment in Idaho for neck and arm pain thought to be caused by a recent work-related injury. On October 19, he was evaluated in the emergency department of hospital A for continued neck pain, nuchal muscle spasms, burning sensation in his right arm, and numbness in the palm of his right hand. He had no fever, chills, or other symptoms of infection. Dehydration was a concern because the patient reported he was unable to drink liquids because of severe pain and muscle spasms. The patient received a prescription for a steroid for muscle spasms and decreased sensation in the right arm and was discharged home.

Two days later, on October 20, the patient developed shortness of breath, tachypnea, and lightheadedness and reported he had not been able to sleep for 4 days; he was transported by ambulance to hospital B. The patient continued to have right upper extremity pain and severe esophageal spasms, causing him to refuse oral fluids. Because of his worsening symptoms and acute delirium, he was transferred to hospital C.

On October 21, the patient was intubated for airway protection. His symptoms worsened, with fever to 104.7°F (40.4°C), and he became comatose on October 25. Additional exposure history collected from family members included ownership of two healthy dogs and a healthy horse, and a recent grouse-hunting trip where the patient had dressed and cleaned the birds while wearing gloves. High-dose corticosteroid treatment was initiated for presumed autoimmune encephalitis. Because of refractory seizures beginning on October 26, he was transferred to hospital D on October 28, where steroids were continued. On November 3, an infectious disease physician was consulted at hospital D who noted that the patient’s symptom of spasms when swallowing suggested a possible diagnosis of rabies. When specifically questioned about the patient’s exposure to wild animals, family members reported extensive contact with bats that had occupied the patient’s home in the weeks before illness onset. The physician notified UDOH, which recommended collecting clinical specimens, including skin, saliva, cerebral spinal fluid (CSF), and serum. Rabies PEP was not indicated because of the advanced state of disease (*1*). The patient continued to decline, supportive care was withdrawn, and he died on November 4, 19 days after symptom onset.

On November 7, antemortem specimens (serum, CSF, skin biopsy, and saliva) were sent to CDC for testing. CDC reported detection of rabies immunoglobulin M and immunoglobulin G in the CSF by indirect immunofluorescence assay. Rabies virus neutralizing antibodies were detected in serum (titer = 1:5,400; 43.2 IU/ml) and in CSF (titer = 1:250; 2.0 IU/ml), by rapid fluorescent focus inhibition test. No rabies virus antigen was detected in skin biopsy by direct fluorescent antibody (DFA) test, and no viral RNA was detected in skin and saliva by real-time reverse transcription–polymerase chain reaction (RT-PCR) (*2*,*3*).

CDC confirmed the presence of rabies virus antigen and RNA in postmortem brain stem tissue and cerebellum specimens by DFA and real-time RT-PCR, respectively. Antigenic typing with monoclonal antibodies to the rabies virus nucleoprotein, and phylogenetic sequence analysis indicated that the virus identified in the patient’s specimens was consistent with that of a rabies virus variant associated with Mexican free-tailed bats (*Tadarida brasiliensis*).

## Public Health Response

Once the rabies diagnosis was confirmed, UDOH established an Incident Command System structure to develop and coordinate response activities. The goals of the response were to 1) determine the source of the patient’s infection; 2) identify possible exposure risk to hospital workers, community members, and family members during the patient’s infectious period; 3) coordinate administration of PEP for exposed persons; and 4) educate health care providers and the public about the risk for rabies associated with contact with bats. Public health investigation and response partners included the Central Utah Public Health Department, Utah County Health Department, Salt Lake County Health Department, Utah Public Health Laboratory, Utah Office of the Medical Examiner, Utah Poison Control Center, Idaho Department of Health, and affected health care facilities, with epidemiologic assistance from CDC. Press briefings were held to provide education and awareness to the general public regarding contact with bats, the risk for rabies, and the importance for persons who had contact with the patient to contact a health care provider or local health department to assess their need for PEP.

The patient’s family reported that, beginning in August, a large number of bats had occupied their attic and frequently were found in the living area of the home, particularly in the master bedroom. On multiple occasions, the patient had removed bats from the home with his bare hands, and on one occasion, the patient awoke to find a bat near his head. In September, a dead bat was found on the floor of the bedroom. Despite the substantial bat contact, no bites were noted. Family members were not aware of health issues related to bat exposure and did not recognize the need to receive rabies PEP after touching bats. After rabies was diagnosed, family members who spent time with the patient were provided PEP. After the patient’s death, to prevent further exposures in the home, a professional bat removal company assessed the patient’s home and sealed all openings that posed a threat for future bat colonization.

The patient’s infectious period was estimated to have begun on October 2, 2 weeks before first symptom onset. Because of the prolonged hospitalization before the rabies diagnosis was made and the number of health care entities involved in the patient’s care, UDOH and health care partners conducted an extensive investigation to identify possible exposures during the patient’s infectious period. To efficiently assess potentially exposed health care workers, an online exposure assessment tool, modeled after a tool used in a mass bat exposure response in Virginia (*4*) was developed and distributed to the four affected health care facilities. Responses were collected at UDOH and provided to the health care facilities, which subsequently ensured that exposed employees received PEP according to Advisory Committee on Immunization Practices guidelines (*5*). The affected health care facilities identified and assessed 242 health care workers known to have had some contact with the patient, which included personnel at each hospital facility, emergency medical transport services, and laboratory workers. A total of 126 (52%) of the 242 exposed health care workers completed the online assessment within 72 hours, and 222 (90%) completed it within 12 days. Among the 242 assessed facility-based health care workers with some contact with the patient, 74 (31%) were determined to have been potentially exposed to infectious materials and received PEP; 63 (85%) of the 74 received PEP within 1 week of initial assessment. The chiropractic workers who initially evaluated the patient were surveyed separately using paper assessment forms; none of the workers were found to have been exposed.

In addition to the 242 potentially exposed facility-based health care workers, public health officials also assessed 37 family and community members who had contact with the patient (total persons assessed = 279); 30 (81%) of the 37 family and community members had contact with the patient’s body fluids and received PEP. The PEP supply used during the response was coordinated and administered by health care facilities throughout Utah. All exposed health care workers completed the PEP regimen as scheduled with only one report of an adverse reaction to the rabies vaccine (gastrointestinal illness reported by one health care provider after receipt of the third vaccine dose).

In April 2019, CDC and UDOH conducted focus group discussions with local health departments involved in the response and with health care workers who cared for the patient. The discussions revealed knowledge gaps about human-to-human rabies transmission among health care workers, and rabies prevention among animal control workers and community members. In response, UDOH and CDC delivered a hospital presentation in hospital D, which was broadcast to hospitals A, B, and C and across the health care system to health care workers in urban and rural areas. Posters and fliers describing the risk for rabies associated with bats were distributed by local public health workers to animal health workers, health care facilities, public health offices, and other public locations.

## Discussion

Human rabies deaths are rare in the United States, and early recognition of the disease can reduce the number of health care–associated exposures and ensure timely receipt of PEP (*3*). Considerations for early recognition include providing education to medical providers (especially those in rural areas) regarding clinical symptoms, identifying patient exposures to wild animals such as bats, and emphasizing the importance of PEP if an exposure occurs. In Utah, humans and animals are most likely to be infected with rabies through exposure to bats, the only known rabies reservoir in Utah.* The Mexican free-tailed bat is the most common host species detected in Utah through public health surveillance (42% of all bats) followed by the Big Brown (21%) and the Silver Haired (15%).

During the past 10 years, an average of 95 bats per year were submitted to the Utah Public Health Laboratory for testing, with 15–25 bats found to be rabid; however, this only accounts for bats tested through the state laboratory and does not count all bats in Utah. The delayed diagnosis of rabies in the patient in this report prevented him from receiving any early treatment for rabies and also resulted in potential rabies exposures for 279 persons in multiple settings during the patient’s infectious period. Structured collaboration between public health partners and health care facilities, as well as the use of online exposure assessment, permitted rapid assessment of exposed persons across numerous settings, facilitating timely recommendation and administration of PEP.

**Summary**

**What is already known about this topic?**

Human rabies is preventable by early recognition of exposure and receipt of postexposure prophylaxis (PEP). Bats are the main source of rabies in the United States.

**What is added by this report?**

Delayed recognition of a human rabies case resulted in potential exposure of 279 health care workers and others in Utah. Exposures were evaluated through an online survey; 74 health care workers with likely rabies virus exposures and 30 family and community members who had contact with the patient’s body fluids received PEP.

**What are the implications for public health practices?**

Educating the general public about the risk for rabies through bat exposure and advising health care providers to consider rabies in the differential diagnosis of unexplained neurologic symptoms could reduce exposures.

## Footnotes

*
http://health.utah.gov/epi/diseases/rabies/surveillance/index.html .
